# Electrochemical Deposition and Characterisation of Silver‐Containing Hydroxyapatite Coatings on Zinc: Influence of Silver Addition on the Functional Properties of a Biodegradable Implant System

**DOI:** 10.1155/ijbm/2080076

**Published:** 2026-09-16

**Authors:** Ivana Mojžišová, Radka Gorejová, Evghenii Harea, Tibor Sopčák, Matej Mičušík, Pavlina Jevinová, Kadir Özaltin, Renáta Oriňaková

**Affiliations:** ^1^ Department of Physical Chemistry, Faculty of Science, Pavol Jozef Šafárik University in Košice, Moyzesova 11, Košice 041 54, Slovakia; ^2^ Centre of Polymer Systems, University Institute, Tomas Bata University in Zlín, Třída Tomáše Bati 5678, Zlín 76001, Czech Republic, utb.cz; ^3^ Institute of Materials Research, Slovak Academy of Sciences, Watsonova 47, Košice 040 01, Slovakia, sav.sk; ^4^ Institute of Polymers, Slovak Academy of Sciences, Bratislava 845 41, Slovakia, sav.sk; ^5^ Department of Food Hygiene, Technology and Safety, University of Veterinary Medicine and Pharmacy, Komenského 73, Košice 041 81, Slovakia, uvm.sk

**Keywords:** Ag-containing coating, bacterial inhibition, electrodeposition, hydroxyapatite

## Abstract

Zinc‐based materials are promising candidates for biodegradable orthopaedic implants; however, their limited bioactivity and insufficient antibacterial performance restrict broader clinical application. Although Ag‐containing hydroxyapatite (HA) coatings have been extensively investigated, most studies have focused on chemically stable substrates, while considerably less attention has been paid to highly reactive biodegradable zinc, for which the effects of silver addition remain insufficiently understood. Therefore, this study investigated how the introduction of silver influences a previously optimised electrochemically deposited hydroxyapatite coating system on zinc. Silver‐containing hydroxyapatite (HA–Ag) coatings were prepared by electrochemical deposition and characterised in terms of morphology, chemical composition, phase structure, corrosion behaviour, tribological performance and preliminary biological response. The applied deposition conditions resulted in the formation of a porous and dendritic HA‐Ag coating with an average thickness of 45 ± 6.8 μm. SEM and AFM analyses revealed increased surface roughness and dendritic morphology of the coating. XRD, FTIR, EDX and XPS results confirmed the formation of calcium phosphate phases together with the presence of silver‐containing species within the coating. The obtained results suggest that silver was present both as metallic particles and in oxidised form associated with the hydroxyapatite layer. Electrochemical measurements performed in Hank’s solution indicated a reduced corrosion rate of HA‐Ag‐coated samples compared to bare zinc. Tribological testing demonstrated altered friction and wear behaviour due to the presence of the ceramic coating. Platelet adhesion assay revealed predominantly spherical platelet morphology, suggesting limited platelet activation under the tested conditions. Antibacterial test performed using the disc diffusion method showed pronounced inhibition zones against *E. coli* and *S. aureus* for the HA‐Ag‐coated samples. Overall, this study contributes to a better understanding of Ag‐containing hydroxyapatite coatings formed directly on biodegradable zinc and demonstrates that introducing silver into a previously optimised hydroxyapatite coating system influences not only the coating characteristics but also the overall coating–substrate system, as evidenced by the formation of AgZn and AgZn_3_ intermetallic phases. Nevertheless, further in vitro and in vivo studies are required to comprehensively assess the biomedical applicability of the developed coating system.

**Highlights**
• Silver‐containing hydroxyapatite coating was successfully deposited on biodegradable zinc substrate by electrochemical deposition method.
• The prepared HA‐Ag coating exhibited porous dendritic morphology with an average thickness of ∼45 μm.
• XRD, FTIR, EDX and XPS analyses confirmed the formation of a calcium phosphate coating containing silver species together with AgZn and AgZn_3_ intermetallic phases.
• The HA‐Ag coating reduced zinc corrosion rate and altered its tribological behaviour under sliding conditions.
• Preliminary antibacterial screening demonstrated diffusion‐mediated inhibition effects against *E. coli* and *S. aureus*, while platelet adhesion experiments suggested limited platelet activation.

## 1. Introduction

With the advancements in modern lifestyle and increasing life expectancy, millions of patients worldwide suffer from bone fractures resulting from injuries, traffic accidents or musculoskeletal disorders, such as osteoporosis, scoliosis or bone cancer [1, 2]. In recent decades, this has led to a growing demand for temporary orthopaedic implants capable of complete degradation within the body after bone regeneration, without adverse effects on the human organism [3–5]. The primary advantage of temporary orthopaedic implants is the elimination of the need for secondary surgical removal, thereby reducing the risk of infection, recurrent fracture or bone mass loss associated with the stress shielding phenomenon [6–8]. Currently, extensive research is dedicated to the development of biodegradable orthopaedic implants based on metals. Compared to polymeric and ceramic alternatives, biodegradable metallic materials exhibit superior mechanical properties, making them particularly suitable for load‐bearing applications [9–11]. Additionally, their ductility allows for processing through various manufacturing techniques [12–15].

Among biodegradable metals, magnesium, iron and zinc are considered the most promising candidates, with zinc receiving the most attention because of its favourable degradation behaviour and biocompatibility [16–18]. All these metals, though, have advantages and limitations. Despite its excellent biocompatibility, magnesium exhibits a rapid degradation rate, which is accompanied by the release of gaseous hydrogen, resulting in the formation of gas bubbles [16], which can interfere with the healing process and threaten the stability of the implant. Iron, however, degrades excessively slowly, which results in the accumulation of bulky corrosion products that are difficult for the body to resorb and may hinder tissue regeneration [17]. Zinc, with an electrode potential of 0.762 V, which is between the electrode potential of magnesium and iron, offers a moderate rate of degradation that aligns more closely with the bone healing process [18–20].

In addition to efforts to develop a biodegradable material that is biocompatible, possesses suitable mechanical properties and exhibits a corrosion rate matching the bone tissue healing process, implant‐associated infections remain a significant challenge in the field of orthopaedic implants [21]. The biogenic nature of implant surface promotes bacterial adhesion, colonisation and the formation of resistant biofilms, which can lead to severe complications, such as implant failure, chronic inflammation, osteomyelitis, amputation or, in extreme cases, death [22, 23]. This issue continues to be one of the major complications in modern orthopaedic surgery [24]. One of the conventional approaches to treating implant‐associated infections is systemic antibiotic therapy. However, this method has significant limitations, including the need for high antibiotic doses due to poor drug efficacy at the site of infection, which can result in hepatorenal or systemic toxicity. Insufficient distribution of antibiotics to the infected area is primarily caused by the limited blood supply to the bones [25]. To address this challenge, researchers are exploring strategies for implant surface modifications and the development of localised drug delivery systems as potential solutions [26–29].

Surface modification with coatings containing antimicrobial agents represents the most promising strategy to prevent implant‐associated infections, as this approach enables localised drug delivery at the site of infection in higher concentrations, thereby reducing systemic toxicity and shortening treatment duration [25, 30].

Among bioactive coatings, phosphate‐based ceramic coatings, such as hydroxyapatite (HA), are currently being extensively studied for orthopaedic implant applications because of their chemical composition and crystallographic structure, which closely resemble native bone. The presence of these compounds on the implant surface enables direct bonding between the implant and bone tissue, thereby promoting osteointegration and bone tissue regeneration [19]. In vivo, HA does not exist in its pure form; instead, calcium ions are substituted by ions present in body fluids, leading to crystal destabilisation and enhanced dissolution [31]. The dissolution rate of HA plays a crucial role in its ability to support the formation of a new bone matrix because faster dissolution facilitates more rapid bone formation at the bone–implant interface [32]. Owing to its exceptional biocompatibility, osteointegration and osteoconductive properties, HA is considered one of the most promising materials for implant coating [33].

Numerous studies have focused on the incorporation of antibiotics into the polymeric or bioceramic coatings; however, organic antimicrobial agents pose a higher risk of inducing bacterial resistance [34, 35], which represents a serious issue in orthopaedic implant‐associated infections. For this reason, the use of inorganic antimicrobial agents is considered a more suitable alternative. Among inorganic substances with antimicrobial properties, silver and its ions are the most well‐known, exhibiting strong inhibitory and bactericidal effects [36]. The reason why silver ions (Ag^+^) induce less likely bacterial resistance is their multiple mechanisms of action, including nucleic acid and protein damage, increased membrane permeability, enzyme inactivation and disruption of the respiratory chain and DNA replication, ultimately leading to microbial cell death [37].

Various methods have been studied for the synthesis of silver‐containing hydroxyapatite, including coprecipitation [38], microwave‐assisted synthesis [39], sol‐gel method [40], hydrothermal method [41] and electrodeposition [42]. Among all methods, only the plasma spraying technique has been clinically approved so far; however, this method has certain limitations, including the extremely high application temperature, which affects the uniformity of the coating and alters the structure of metallic implants [43]. Electrochemical deposition is considered a more suitable method, as it is a cost‐effective and straightforward process that can be applied even to complex substrates. Compared to conventional coating approaches, electrochemical deposition enables direct surface modification under relatively mild processing conditions and allows control over coating growth, composition and morphology. These features make the method particularly attractive for the preparation of multifunctional calcium phosphate–based coatings on biodegradable zinc substrates [44].

Although hydroxyapatite and Ag‐containing hydroxyapatite coatings have been extensively investigated, most studies have focused on chemically stable substrates, particularly titanium and its alloys. However, biodegradable zinc represents a substantially different material due to its high chemical activity, dynamic oxidation/passivation behaviour and challenges associated with stable coating nucleation, homogeneous surface coverage and coating adhesion during electrochemical processing. Consequently, knowledge obtained from titanium‐based systems cannot be directly extrapolated to zinc substrates.

In our previous study [45], the electrochemical deposition conditions for hydroxyapatite coating formation on zinc were optimised using EDTA‐assisted electrodeposition. Building upon these findings, an important question arises: How does the introduction of silver influence the properties of such an established coating system?

Therefore, the present work aims to prepare and characterise silver‐containing hydroxyapatite coatings directly deposited on zinc and to systematically evaluate how silver addition influences coating morphology, surface chemistry, corrosion behaviour, tribological performance and preliminary biological response under identical deposition conditions.

## 2. Materials and Methods

### 2.1. Preparation of Zinc Samples

The zinc samples were prepared from raw zinc powder (99.5% purity, Centralchem, Slovakia) by the powder metallurgy method. Zinc powder was compressed using a hydraulic press (Redats H‐380, P.H.U Szczepan, Krakow, Poland) at 745 MPa into the pellets (Ø 13 mm) and sintered in a tube furnace (Nabertherm 30–3000, Nabertherm GmbH, Germany) at 350°C for 1 h under an inert atmosphere of argon (flow rate of 4 L/min). Subsequently, the sintered samples were cooled to laboratory temperature.

### 2.2. Electrochemical Deposition of Silver‐Containing Hydroxyapatite

Before the hydroxyapatite coating deposition, the entire surface of the samples was gradually sanded with two different grits of SiC paper (P600 and P1200). The samples were then ultrasonically cleaned for 10 min in acetone (Centralchem, Slovakia) and 10 min in 96% ethanol (Centralchem, Slovakia).

The silver‐containing hydroxyapatite coating (HA‐Ag) was electrochemically deposited from a solution containing 3.78·10^−2^ mol/dm^3^ Ca(NO_3_)_2_·4H_2_O, 0.42·10^−2^ AgNO_3_, 2.5·10^−2^ mol/dm^3^ NH_4_H_2_PO_4_ and 7.5·10^−4^ mol/dm^3^ EDTA‐2Na [46] to maintain a (Ca + Ag)/P ratio corresponding to the stoichiometric Ca/P ratio in pure hydroxyapatite. Electrochemical deposition was carried out in galvanostatic mode using a potentiostat (Autolab Multichannel M204, Metrohm, Switzerland) in a three‐electrode system at a current density of 0.4 mA/cm^2^ and a constant temperature of 65°C for a duration of 120 min. The zinc pellet was used as the working electrode, a platinum sheet as the counter electrode and an Ag/AgCl/KCl (3 mol/dm^3^) as the reference electrode.

Based on studies demonstrating that the electrochemically deposited coating is predominantly in the form of dicalcium phosphate dihydrate (DCPD), which can be transformed into the hydroxyapatite through thermal alkali treatment [47–49], the coated samples were immersed in 1 mol/dm^3^ NaOH at 65°C for 2 h, followed by washing in distilled water and drying at 80°C for 2 h.

### 2.3. Samples Characterisation

The Zn–HA and Zn–HA–Ag samples were prepared and tested within the same experimental series using identical testing procedures, with Zn–HA serving as the Ag‐free reference coating. The detailed polarisation, tribological and platelet adhesion results for Zn–HA were reported previously as part of the optimisation study of this coating system [45]. Therefore, these results are not presented again in full in the corresponding sections of the present manuscript but are referenced and summarised in disucssion to enable comparison with Zn–HA–Ag.

#### 2.3.1. Morphological Analysis

The surface morphology of the coated and uncoated samples was analysed by optical microscopy (Dino‐Lite AM4815ZT and Dino‐Lite AM4515T8, ∼20–900 × magnification, 1.3 MPx, Dino‐Lite, Delmenhorst, The Netherlands) and scanning electron microscopy (JEOL JSM‐7000F, Japan). The surface topology of the samples was observed using a Dimension Icon atomic force microscope (AFM, Bruker, Karlsruhe, Germany) in a tapping mode with a ScanAsyst‐Air Si/Nitride probe (Bruker, Santa Barbara, CA, USA). The spring constant of the cantilever was *k* = 0.4 N/m. Scanning was conducted over an area of 1 × 1 μm at a frequency of 1 Hz for each sample. For the average surface roughness (*R_a_*) value analysis, NanoScope Analysis software was used.

#### 2.3.2. Chemical and Phase Analysis

The elemental analysis of the sample surface was performed using energy‐dispersive X‐ray spectroscopy (EDX, INCA analyser, Japan), which provided information about the elemental distribution and their relative representation on the surface. The characteristic functional groups of the HA‐Ag coating were analysed using Fourier transform infrared spectroscopy (FTIR) with an IRTracer‐100 infrared spectrometer (Shimadzu, Japan). The FTIR spectrum was measured three times from different locations of the samples using the attenuated total reflection (ATR) method. The phase composition of the HA‐Ag coating was analysed using an X‐ray diffractometer (XRD, Philips X’Pert Pro, Germany) equipped with Co Kα radiation (*λ* = 1.789 Å) operated at 40 kV and 50 mA in the scanning range of 10–110° (2*θ*). X‐ray diffraction was performed on the surface of the samples. In addition, the hydroxyapatite collected after the electrochemical deposition of the ceramic coating, which was not subjected to thermal alkali treatment, was also examined. The obtained hydroxyapatite was calcinated at 360°C for 1 h under an atmosphere. The chemical composition and oxidation state of the sample surface were determined using X‐ray photoelectron spectroscopy (XPS, Thermo Fisher Scientific, UK) using a Thermo Scientific NEXSA‐G2 XPS system equipped with a microfocused, monochromatic Al Kα X‐ray source (1486.68 eV) and 400 μm X‐ray beam. Measurements were performed in the constant analyser energy mode. Survey spectra were acquired using a pass energy of 200 eV, while high‐resolution (HR) spectra were collected using a pass energy of 50 eV. The energy step size was set to 1 eV for survey scans and 0.1 eV for narrow regions. Surface charging effect was compensated using an Ar^+^ flood gun. Data acquisition and processing were carried out using Thermo Scientific Avantage software (Version 6.10.0, Thermo Fisher Scientific).

#### 2.3.3. Mechanical Analysis

To assess the surface mechanical properties of the samples, a dry sliding friction test was conducted with the Bruker Universal Mechanical Tester (UMT). The instrument was operated in the linear ball‐on‐flat reciprocating mode, which allows the evaluation of tribological behaviour under controlled contact and movement conditions. Measurements were performed under room temperature and air conditions. The tests were performed with a relative humidity of 48 ± 5%. A 3 mm stainless steel ball served as the sliding counterface against the flat specimen surface. Stainless steel ball counterbody enables accurate durability analysis of the ceramic coating due to its superior hardness which minimises reciprocal wear. Experiments were carried out under a constant normal load of 0.05 N, with a 5 mm stroke, for a total of 250 cycles lasting 250 s.

#### 2.3.4. Corrosion Property Evaluation

The degradation properties of the prepared materials were electrochemically studied using linear sweep voltammetry (LSV) at a constant physiological temperature of 37 ± 2°C in Hank’s solution simulating human extracellular fluids with a pH of 7.4 ± 0.2 composed of 8 g/L NaCl, 0.4 g/L KCl, 0.14 g/L CaCl_2_, 0.06 g/L NaH_2_PO_4_·2H_2_O, 0.35 g/L NaHCO_3_, 0.06 g/L MgSO_4_·7H_2_O, 1.00 g/L glucose, 0.10 g/L MgCl_2_·6H_2_O and 0.60 g/L KH_2_PO_4_. The LSV measurements were performed using a potentiostat (Autolab Multichannel M204, Metrohm, Switzerland) in a three‐electrode setup, where the studied samples served as the working electrode, an Ag/AgCl/KCl (3 mol/dm^3^) electrode as the reference electrode and a Pt sheet as the counter electrode. Before the measurements, the uncoated samples were ultrasonically cleaned in acetone and 96% ethanol, each for 10 min. The system was stabilised for 60 min before each measurement, and the open circuit potential (OCP) value was recorded. Subsequently, LSV was performed three times. During the measurements, the samples were placed in a corrosion cell sample holder (CORR.1LHLD, Metrohm, Switzerland) to ensure a precisely defined measured area size of 1 cm^2^. The potentials were scanned at a rate of 0.1 mV/s.

From the obtained Tafel curves, corrosion current values were determined using the Tafel extrapolation method. These values were then used to calculate the degradation rate, following Equation (1) in accordance with ASTM G59 [50]:

(1) CR=jcorr K EWρ

where CR represents the corrosion rate (mm/year), *j*_corr_ is the corrosion current density (μA/cm^2^), *K* is the constant used to convert the units of CR from the current density, with a value of 3.27 × 10^−3^, EW is the equivalent weight of the corroding material, and *ρ* is the material density (g/cm^3^). Although the coated samples contained a HA‐Ag layer, the coating itself is not expected to undergo significant electrochemical dissolution under the applied testing conditions. Due to the fact that the measured corrosion current is assumed to be predominantly associated with the electrochemical degradation of the zinc substrate, the equivalent weight corresponding to zinc (32.69 g/eq) was used for both coated and uncoated samples. The HA‐Ag coating acts primarily as a surface modification layer affecting the corrosion process rather than as the principal corroding phase. Therefore, corrosion rates were calculated with respect to the underlying zinc substrate. A similar approach has been reported for hydroxyapatite‐coated zinc substrates, where corrosion rates were calculated using the equivalent weight and density of zinc despite the presence of a surface coating [51].

To further investigate the corrosion properties of the prepared samples, electrochemical impedance spectroscopy (EIS) was employed. The EIS measurements were carried out using a potentiostat (Autolab Multichannel M204, Metrohm, Switzerland) with the same three‐electrode setup as that used during LSV. The measurements were performed in Hank’s solution at a constant temperature of 37 ± 2°C over a frequency range from 100 kHz to 10 Hz with an AC amplitude of 10 mV. All measurements were performed in triplicate to ensure reproducibility.

#### 2.3.5. Surface Analysis After Corrosion

XRD analysis was conducted after immersion in Hank’s solution to identify crystalline corrosion products and evaluate phase evolution during the degradation process. Zn‐HA and Zn‐HA‐Ag samples were immersed in Hank’s solution at 37 ± 2°C for 2, 4 and 8 h. In addition, a bare Zn sample was analysed after 8 h of immersion as a reference representing the corrosion behaviour of the uncoated substrate. The primary objective of this experiment was to compare the phase evolution of the coated samples and evaluate whether the presence of silver influenced the nature of the corrosion products formed during immersion. After exposure, the samples were rinsed with distilled water, air‐dried and analysed under the same XRD conditions described in Section 2.3.2.

#### 2.3.6. Biological Property Evaluation

##### 2.3.6.1. Platelet Adhesion Assay

Platelet adhesion on the prepared samples was evaluated using a platelet adhesion assay with reference to ISO 10993‐4 as a preliminary qualitative assessment of platelet–surface interactions. Whole blood was obtained from a single experimental rat under a protocol approved by the State Veterinary and Food Administration of the Slovak Republic under the number 5999‐3/2023‐220. One specimen from each material group (Zn and Zn–HA–Ag) was evaluated using platelet‐rich plasma prepared from this blood sample. Five distinct surface areas were examined by SEM on each specimen to assess platelet adhesion and morphology. Before testing, samples were incubated in PBS at 37°C for 30 min, rinsed with distilled water and air‐dried. After that, the samples were disinfected under germicidal UV light for 30 min. Platelet‐rich plasma was obtained by centrifuging whole blood at 1600 rpm for 15 min. The samples were transferred to 24‐well plates, and 1 mL of plasma was added to each sample, followed by incubation at 37°C for 2 h. Nonadherent platelets were then removed by rinsing with PBS, while adherent platelets were fixed on the surface using 2.5% glutaraldehyde for 30 min at laboratory temperature. To stabilise the adhered cells, samples were dehydrated in graded ethanol solutions (50%, 60%, 70%, 80%, 90% and 100%) for 10 min at 4°C, followed by freeze‐drying. The surface of the samples was examined by scanning electron microscopy (SEM, JEOL JSM‐7000F, Japan).

##### 2.3.6.2. Disc Diffusion Antibacterial Assay

`The diffusion‐mediated inhibition zone formation of the Zn disc samples was preliminarily evaluated using the disc diffusion method (Kirby–Bauer assay, [52]) against two common bacterial strains, *Escherichia coli* CCM 3954 and *Staphylococcus aureus* CCM 4223 (Czech Collection of Microorganisms, Brno, Czech Republic). In a typical procedure, 100 μL aliquots of standardised bacterial suspensions, adjusted to 0.5 McFarland turbidity, were uniformly spread onto the surface of predried agar plates using a sterile L‐shaped spreader (HiMedia, India). Baird–Parker agar was used for *S. aureus*, while Endo agar was used for *E. coli*. After inoculation and absorption of the bacterial suspension, the Zn discs were carefully placed onto the agar surface under sterile conditions. The inoculated plates were incubated aerobically at 37°C for 24 h. After incubation, the inhibition effect was assessed by measuring the diameter of the inhibition zones, including the disc diameter, using a digital calliper (MITUTOYO, Japan). In addition, the width of the inhibition zone measured from the edge of the disc was recorded to better reflect the diffusion efficiency of antibacterial species into the agar medium. All measurements were performed in triplicate and are reported as mean values ± standard deviation. The disc diffusion assay was intended as a qualitative evaluation of diffusion‐mediated inhibition effects of the prepared samples.

## 3. Results

### 3.1. Surface Morphology of Zn and Zn‐HA‐Ag Samples

**FIGURE 1:**
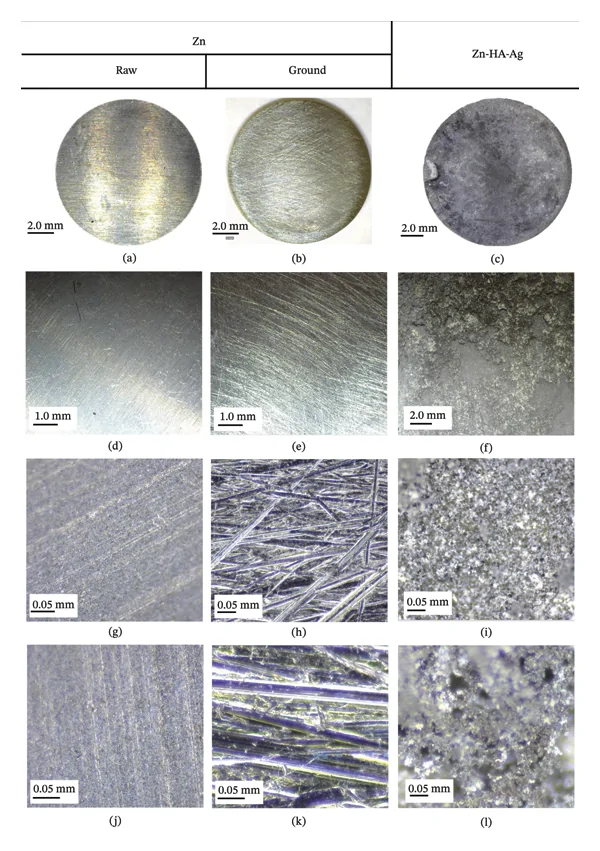
Optical microscopy images of (a, d, g, j) Zn before and (b, e, h, k) after grinding and (c, f, i, l) Zn coated with Ag‐containing hydroxyapatite.

Figure 1 displays optical images of the surfaces of Zn and Zn‐HA‐Ag. Before grinding and the deposition of the ceramic coating, the surface of the Zn pellets exhibited smooth texture (Figure 1a,d) with discernible striping characterised by alternating lighter and darker bands (Figure 1g,j). The surface of the samples was ground with SiC papers to increase surface roughness and improve ceramic coating adhesion, resulting in grooves of varying sizes oriented in different directions (Figure 1e,h,k). The bluish appearance observed in some grinding grooves is attributed to light reflection effects caused by the surface topography.

The electrochemical deposition of the silver‐containing hydroxyapatite led to the coating evenly distributed over the entire surface of the sample, consisting of crystals with a metallic lustre. The predominant colour of the coating was grey, with variations including darker and lighter areas. The thickness of the coating was not uniform, with certain regions of the coating exhibiting thinner layers.

**FIGURE 2:**
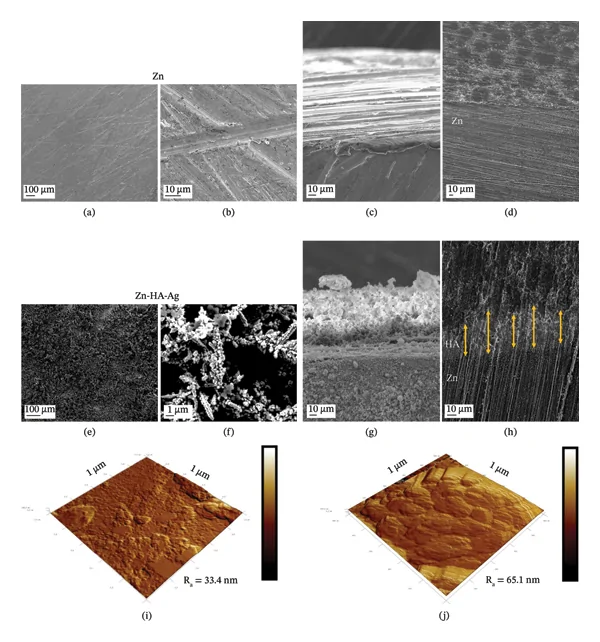
SEM images of the surfaces of (a, b) Zn and (e, f) Zn‐HA‐Ag samples. Lateral views of the surfaces of (c) Zn and (g) Zn‐HA‐Ag. Cross‐sectional views of (d) Zn and (h) Zn‐HA‐Ag. AFM graphs of (i) Zn and (j) Zn‐HA‐Ag.

Upon closer inspection at higher magnification, the SEM images of Zn (Figure 2a,b) and Zn‐HA‐Ag coating (Figure 2e,f) revealed that the Zn‐HA‐Ag coating consists of branch‐like structures composed of agglomerated particles forming globular clusters. The average size of the globular particles was 0.59 ± 0.11 μm. The considerable complexity of the coating resulted in a large surface area and the formation of a porous dendritic structure. Figure 2 displays the lateral view of Zn (Figure 2d) and Zn‐HA‐Ag (Figure 2g) samples, illustrating the growth pattern of the ceramic coating during electrochemical deposition, which led to an uneven surface. The coating thickness is variable with hydroxyapatite aggregates present in some areas. Figure 2d,h displays the cross‐section of the samples. The average thickness of the coating is 45 ± 6.8 μm (Figure 2h).

The AFM data revealed changes in local surface roughness after coating with Zn‐HA‐Ag (Figure 2i,j). AFM analysis performed over a local 1 × 1 μm scan area showed that the initial surface roughness increased from 33.4 nm for bare Zn to 65.1 nm after coating deposition. The AFM measurements were primarily intended to evaluate nanoscale topographical and roughness changes induced by the coating, while the overall porous and dendritic morphology of the Zn‐HA‐Ag surface was assessed by SEM observations at different magnifications. The final surface morphology and chemical composition of Zn‐HA‐Ag are expected to influence its corrosion behaviour and diffusion‐mediated inhibition response.

### 3.2. Chemical and Phase Composition of Coated Samples

**FIGURE 3:**
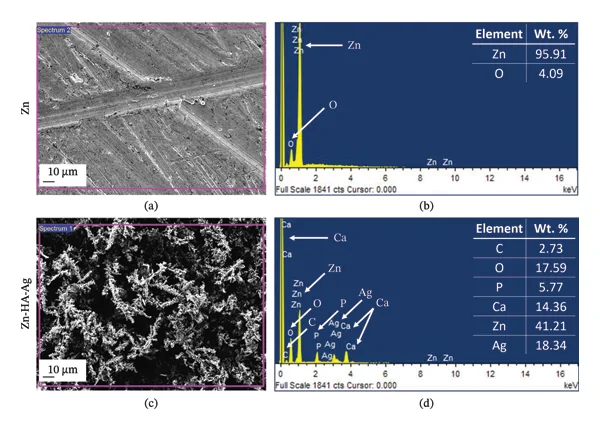
(a, c) SEM images and (b, d) corresponding EDX spectra with elemental compositions of the surfaces of Zn and Zn‐HA‐Ag.

The elemental composition of the surface of the sintered zinc sample and the zinc sample coated with silver‐containing hydroxyapatite was analysed using EDX area analysis (Figure 3). On the surface of the bare zinc sample, EDX detected the presence of oxygen in addition to zinc. In the case of the zinc sample coated with silver‐containing hydroxyapatite, the presence of Ca, P, O, Zn, Ag and C was revealed.

**FIGURE 4:**
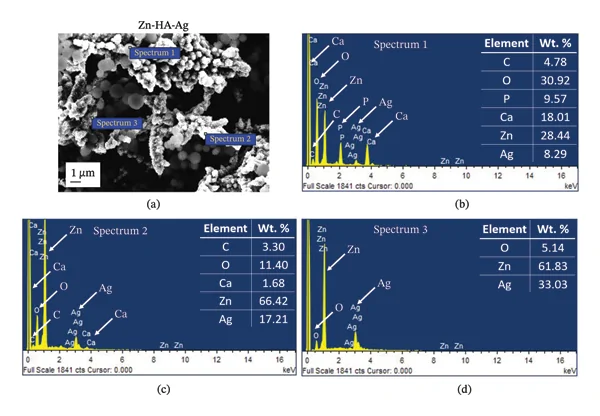
EDX point analysis of the Zn‐HA‐Ag surface: (a) SEM image with the analysed points and (b, c, d) corresponding EDX spectra and elemental compositions.

The surface of the coated samples was further examined using EDX point analysis (Figure 4), which identified the same elements, with zinc and silver prevailing over calcium in most areas of the coating. Based on the elemental composition at the analysed points, slight chemical variations were observed across different surface regions.

**FIGURE 5:**
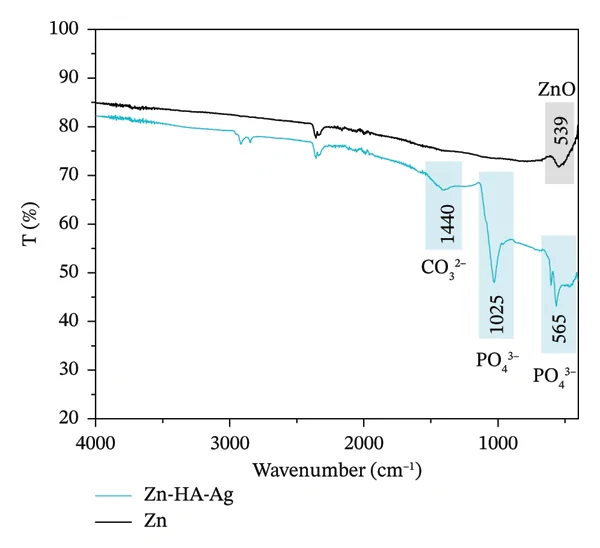
FTIR analysis of the surface of Zn and Zn‐HA‐Ag sample.

The presence of characteristic functional groups in the coating was examined using FTIR, as shown in Figure 5, which compares the FTIR spectra of Zn and Zn‐HA‐Ag samples. The FTIR spectrum of bare Zn exhibits two absorption bands of low intensity at 539 cm^−1^ and in the region of 2358 cm^−1^, while the band at 2358 cm^−1^ is also present in the spectrum of the coated samples. The spectrum of the coated samples displays additional absorption bands at 2919, 2850, 1440, 1025 and 565 cm^−1^, which are discussed below.

**FIGURE 6:**
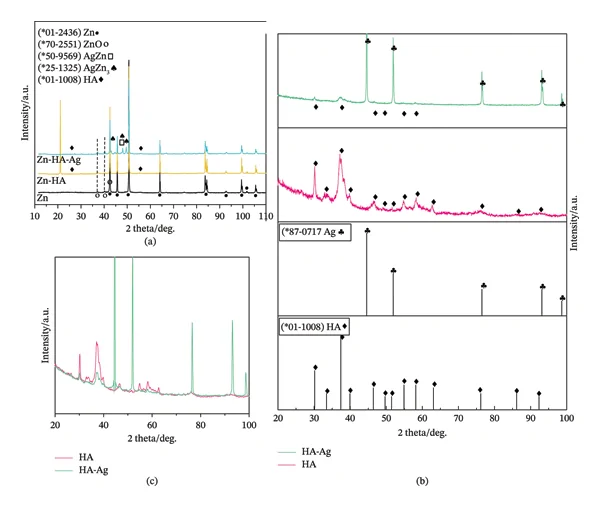
XRD patterns of (a) uncoated Zn and Zn samples coated with HA and HA‐Ag (b) hydroxyapatite and silver‐containing hydroxyapatite obtained by filtration of the electrolyte solution after the electrochemical deposition, without subsequent thermal alkali treatment, together with reference standard patterns, and (c) overlay of HA and HA‐Ag diffraction patterns.

Figure 6 shows the XRD patterns of the prepared samples and coatings separately. In Figure 6a, the diffraction patterns of uncoated Zn, Zn coated with HA‐Ag coating, and, for comparison, the hydroxyapatite coating without silver (HA) from our previous study [45] are shown. The HA coating was used as a reference to evaluate changes in crystallinity and phase composition associated with the addition of silver‐containing species. In all spectra, characteristic diffraction peaks of the Zn substrate were observed at approximately 36°, 39°, 43°, 54°, 70°, 82°, 83° and 86° 2*θ*, corresponding to the standard ICDD 01‐2436. Additional reflections attributed to ZnO were also detected on the Zn surface. In the case of the Zn‐HA‐Ag sample, peaks at ∼37°, 41° and 42° 2*θ* characterised for the intermetallic phases AgZn and AgZn_3_ were observed. The peaks for hydroxyapatite were present with very low intensity. In Figure 6b, the diffraction patterns of pure HA and HA‐Ag coatings are presented, corresponding to the standard reference pattern ICDD 01‐1008 for HA. These hydroxyapatite powders were obtained from the electrolyte solution after electrochemical deposition and were calcinated at 360°C for 1 h. In the case of the HA‐Ag coating, additional reflections characteristic of cubic silver were identified according to ICDD 87–0717. Figure 6c provides a direct comparison of the HA and HA–Ag coatings, highlighting differences in phase composition and crystallinity. The overlay demonstrates that although both coatings exhibit reflections consistent with HA, the characteristic HA peaks are less intense in the HA–Ag coating. However, the presence of Ag diffraction peaks did not lead to significant alterations in the diffraction pattern of HA.

**FIGURE 7:**
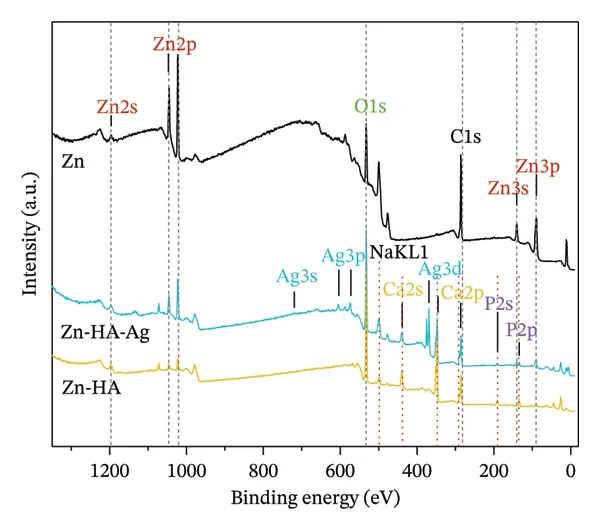
XPS survey spectra of Zn, Zn‐HA‐Ag and Zn‐HA.

The elemental composition and oxidation state of the surface were investigated using XPS. In addition to the Zn and Zn‐HA‐Ag samples, XPS spectra were also acquired for zinc coated with Ag‐free hydroxyapatite (Zn‐HA), similarly to the XRD analysis. Figure 7 shows the survey XPS spectra of Zn, Zn‐HA and Zn‐HA‐Ag samples. In all samples, signals corresponding to Zn, O and C were detected. The coated samples exhibited additional Ca and P peaks, confirming the presence of a Ca‐P‐based layer on the zinc surface. In the case of the Zn‐HA‐Ag sample, Ag‐related peaks were also observed. The presence of these elements was also confirmed by EDX analysis.

**FIGURE 8:**
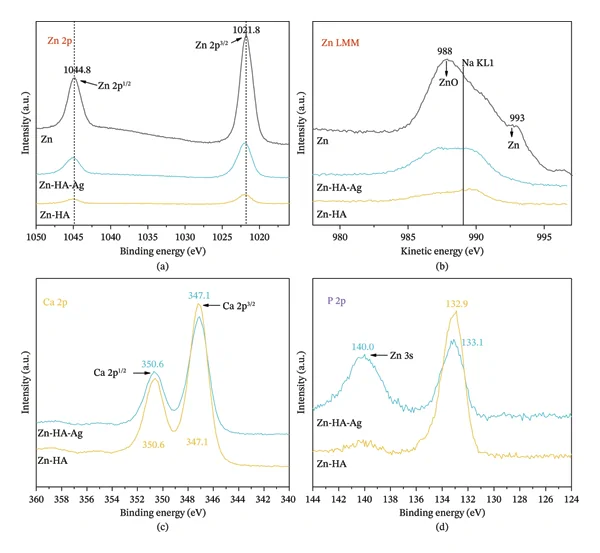
High‐resolution XPS spectra of (a) Zn 2p region, (b) Zn LMM, (c) Ca 2p region and (d) P 2p region.

**FIGURE 9:**
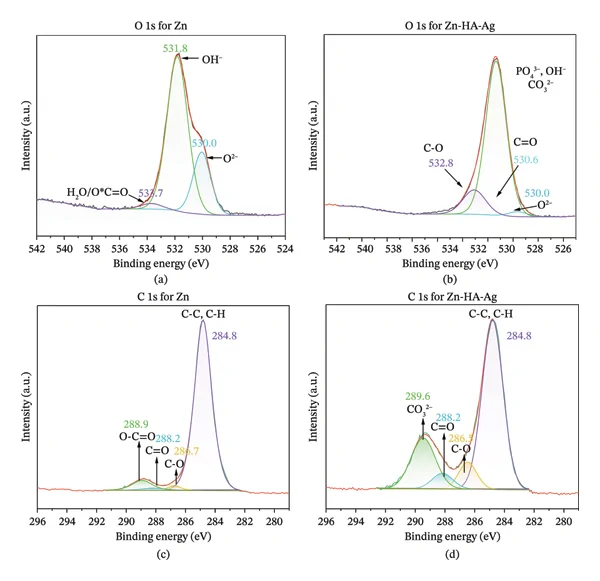
High‐resolution XPS spectra of O 1s region for (a) Zn, (b) Zn‐HA‐Ag and C 1s region for (c) Zn, (d) Zn‐HA‐Ag.

Furthermore, the HR spectra of Zn 2p, Ca 2p, P 2p, O 1s, C 1s and Ag 3d were recorded (see Figure 8 and 9). In Table 1, the peak positions used for particular HR spectral deconvolution are displayed, and in Table 2, quantifications are from these deconvolutions.

**TABLE 1:** XPS binding energy values of the principal elements.

Sample	Binding energy (eV)						
	Zn 2p^3/2^ Zn 2p^1/2^	Zn LMM^∗^	Ca 2p^3/2^ Ca 2p^1/2^	P 2p	O 1s	C 1s	Ag 3d^5/2^ Ag 3d^3/2^
Zn	1021.8	988	—	—	530.1	284.8	—
	*1044.8*	993			531.8	286.7	
					533.7	288.2	
						288.9	

Zn‐HA	1022.0	988	347.1	132.9	530.0	284.8	—
	*1045*.*0*		*350.6*		530.7	286.5	
					531.4	288.2	
					532.8	289.6	

Zn‐HA‐Ag	1022.0	988	347.1	133.1	530.0	284.8	367.7
	*1045*.*0*		*350.6*		530.6	286.5	*373.7*
					531.4	288.2	368.5
					532.8	289.6	*374.5*

^∗^Kinetic energy values.

**TABLE 2:** Atomic concentrations obtained by XPS for bare Zn and coated samples.

Sample	Surface chemical composition (at. %)					
	C1s (COOH/CO_3_^2−^)	O1s	Zn2p	Ag3d Ag^+^/Ag^0^	Ca2p/P2p	N1s/Na1s/Si2p/Cl2p
Zn	59.9 (3.7)	24.4	14.9	—	—/—	0.6/—/—/0.3
Zn‐HA	31.3 (6.9)	46.0	1.6	—	13.4/5.0	—/1.6/1.2/—
Zn‐HA‐Ag	32.3 (7.2)	42.3	5.8	3.1 2.2/0.9	9.6/2.4	0.7/2.3/1.2/0.1

Figure 8a presents the HR Zn 2p spectra, where all samples exhibit two characteristic spin–orbit components corresponding to Zn 2p^3/2^ at 1021.8 eV and Zn 2p^1/2^ at 1044.8 eV. As the Zn 2p region does not allow a reliable distinction between metallic Zn (Zn^0^) and oxidised Zn (Zn^2+^), Zn LMM Auger spectra were obtained (Figure 8b). For detailed discussion of XPS results, see the discussion section below.

### 3.3. Tribological Behaviour of the Sample Surface

**FIGURE 10:**
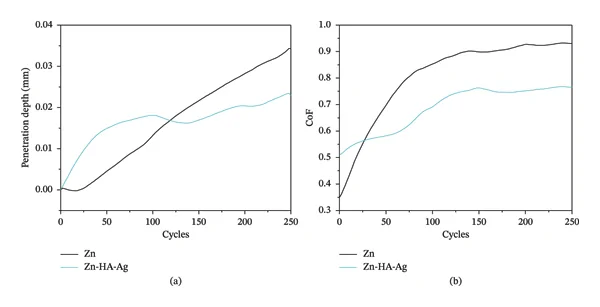
(a) Friction track depth of Zn and Zn‐HA‐Ag (b) values of the coefficient of friction of Zn and Zn‐HA‐Ag.

The tribological response of the investigated samples was assessed via reciprocating friction tests employing a stainless steel ball as the counterbody. Figure 10 presents the evolution of penetration depth and the corresponding progression of the coefficient of friction (CoF) throughout the test duration. Because penetration depth is geometrically related to wear‐track volume under the applied ball‐on‐flat test geometry, it can be used as an indirect indicator of wear behaviour.

For the Zn‐HA‐Ag sample (Figure 10a), the penetration depth increased rapidly during the initial 30–40 sliding cycles. It subsequently continued to increase at a lower rate, reaching a local maximum of approximately 0.018 mm after about 100 cycles. Between approximately 100 and 140 cycles, the penetration depth decreased slightly to approximately 0.016 mm, after which it gradually increased and reached approximately 0.023 mm at the end of the test.

In contrast, the uncoated zinc substrate demonstrated an anomalous ‘negative wear’ phenomenon within the first 5–8 cycles, discussed below. Subsequently, a gradual increase in penetration depth was recorded until the end of test. The crossover point at which the coated system exhibited lower penetration depth than the bare substrate was observed after approximately 110–120 cycles.

The CoF responses are presented in Figure 10b. The uncoated zinc sample exhibited a progressive increase in CoF from approximately 0.35 at the beginning of the test to approximately 0.90 after 140–150 cycles, followed by only minor changes until the end of the test. The Zn‐HA‐Ag sample initially exhibited a higher CoF of approximately 0.50. Its CoF increased more gradually and remained lower than that of the uncoated substrate from approximately 20–30 cycles until the end of the test.

### 3.4. Corrosion Behaviour

#### 3.4.1. Potentiodynamic Polarisation Measurement

**FIGURE 11:**
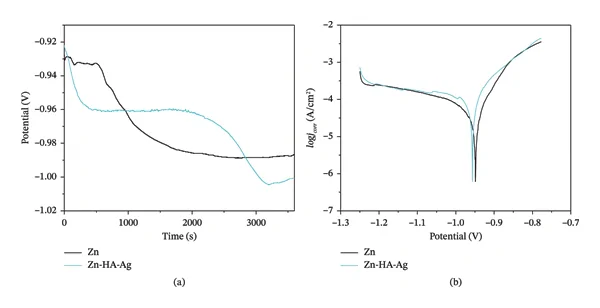
Representative potentiodynamic polarisation curves of Zn and Zn‐HA‐Ag measured in Hank’s solution.

The OCP measured during system stabilisation is presented in Figure 11 as an OCP time plot for both uncoated and coated samples. Both samples exhibited a similar initial OCP value of approximately −0.93 V. After 520 s, OCP potential of Zn continually decreased and stabilised at the potential value of −0.99 V within 2000 s. In the case of zinc coated with silver‐containing hydroxyapatite, the OCP potential value stabilised after 400 s at the potential of −0.96 V and after 2000 s significantly decreased to the potential of −1.00 V. The coated sample stabilised at the more negative potential (Figure 11a).

Figure 11b presents the representative potentiodynamic polarisation curves of uncoated and coated zinc samples recorded in Hank’s solution. The corresponding corrosion parameters, including corrosion potential, corrosion current density and corrosion rate, obtained from polarisation curves using Tafel extrapolation, are summarised in Table 3. The corrosion potential was −0.96 ± 0.03 V for the uncoated zinc sample and −0.97 ± 0.02 V for the coated sample. Compared to the polarisation curve of bare zinc, the silver‐containing hydroxyapatite coating caused a shift in corrosion potential towards more negative values (Figure 11b).

**TABLE 3:** Electrochemical parameters obtained by Tafel extrapolation from polarisation curves for uncoated and coated zinc sample.

	Zn	Zn‐HA‐Ag
*E*_corr_ (V)	−0.96 ± 0.03	−0.97 ± 0.02
*j*_corr_ (A/cm^2^)	3.52·10^−5^ ± 1.37·10^−6^	2.72·10^−5^ ± 1.88·10^−6^
Corrosion rate (mmpy)	0.52 ± 0.02	0.42 ± 0.03
Polarisation resistance (Ω)	290.28 ± 9.18	401.31 ± 137.82

However, the Zn‐HA‐Ag sample simultaneously exhibited lower calculated corrosion current density values (3.52·10^−5^ ± 1.37·10^−6^ A/cm^2^ for Zn and 2.72·10^−5^ ± 1.88·10^−6^ A/cm^2^ for Zn‐HA‐Ag) together with lower calculated corrosion rate values derived from Tafel extrapolation (0.52 ± 0.02 mm/year for Zn and 0.42 ± 0.03 mm/year for Zn‐HA‐Ag). In addition, the polarisation resistance increased from 290.28 ± 9.18 Ω for bare Zn to 401.31 ± 137.82 Ω for the coated sample. These results suggest that the corrosion behaviour of the HA‐Ag‐coated system involves competing electrochemical processes and cannot be interpreted solely as a simple passive barrier effect.

#### 3.4.2. Impedance Measurement

**FIGURE 12:**
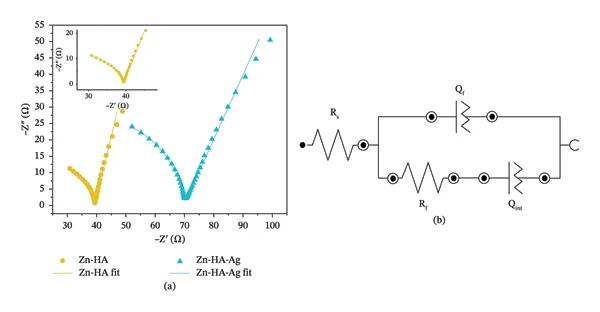
(a) The fitted Nyquist plots of Zn‐HA and Zn‐HA‐Ag, (b) the equivalent electrical circuit used to fit EIS data of coated samples.

The fitted electrochemical impedance spectra of coated Zn‐HA and Zn‐HA‐Ag samples measured in Hank’s solution are presented in Figure 12a. To improve the visualisation of the Zn‐HA specimen, a magnified view of the low‐impedance region is shown. A shift of the impedance response towards higher *Z* ^′^ values was observed after silver incorporation into the hydroxyapatite coating.

The impedance spectra of Zn‐HA and Zn‐HA‐Ag were successfully fitted using the equivalent electrical circuit as shown in Figure 12b. The equivalent circuit consisted of the solution resistance (*R*_*s*_), a constant phase element associated with the coating layer (*Q*_*f*_), the coating resistance (*R*_*f*_) and a second constant phase element (*Q*_int_) describing the interfacial electrochemical response. The parameters obtained from the fitting procedure are summarised in Table 4.

**TABLE 4:** The values of the equivalent electrical circuit’s parameters of coated samples.

	Zn‐HA	Zn‐HA‐Ag
*R*_*s*_ (Ω)	9.41 ± 0.65	11.19 ± 0.50
*R*_*f*_ (Ω)	29.92 ± 0.74	58.48 ± 0.70
*Q*_*f*_ (Ω^−1^·s^n^)	1.40·10^−7^ ± 6.65·10^−8^	4.33·10^−8^ ± 3.75·10^−9^
*n*_*f*_	0.88 ± 0.01	0.93 ± 0.01
*Q*_int_ (Ω^−1^·s^n^)	0.11·10^−2^ ± 3.82·10^−5^	0.001 ± 0.11·10^−3^
*n*_int_	0.82 ± 0.01	0.68 ± 0.02
*χ*2	0.004 ± 0.9·10^−3^	0.005 ± 0.002

The fitted *R*_*s*_ values were 9.41 ± 0.65 Ω and 11.19 ± 0.50 Ω for Zn‐HA and Zn‐HA‐Ag, respectively. The resistance associated with the coated surface (*R*_*f*_) increased from 29.92 ± 0.74 Ω for Zn‐HA to 58.48 ± 0.70 Ω for Zn‐HA‐Ag. The exponent *n*_*f*_ increased from 0.88 ± 0.01 to 0.93 ± 0.01, while *Q*_*f*_ decreased from 1.40·10^−7^ ± 6.65·10^−8^ to 4.33·10^−8^ ± 3.75·10^−9^ Ω^−1^·s^n^ following Ag incorporation. The *Q*_int_ values were 0.11·10^−2^ ± 3.82·10^−5^ and 0.001 ± 0.11·10^−3^ Ω^−1^·s^n^ for Zn‐HA and Zn‐HA‐Ag, respectively, whereas the corresponding *n*_int_ values decreased from 0.82 ± 0.01 to 0.68 ± 0.02. The *χ* ^2^ values remained below 0.01 for both fitted spectra, indicating a satisfactory agreement between the experimental and simulated data.

#### 3.4.3. Postcorrosion Phase Analysis

**FIGURE 13:**
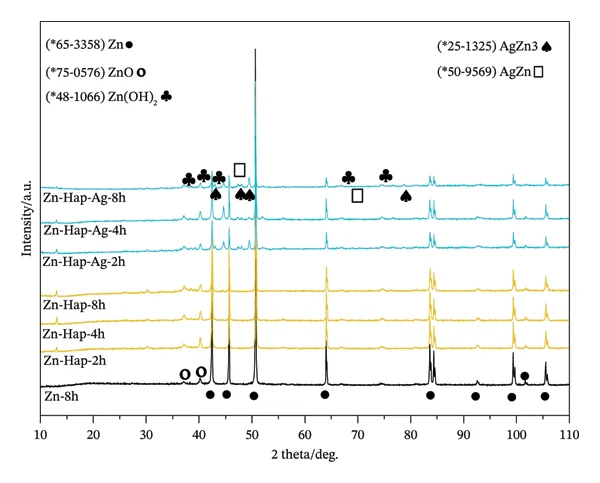
XRD analysis of the surface of Zn after 8 h and Zn‐HA and Zn‐HA‐Ag after 2, 4 and 8 h of immersion in Hank’s solution.

To further investigate the corrosion behaviour of the coated samples, XRD analysis was performed after immersion in Hank’s solution for 2, 4 and 8 h (Figure 13). Compared with the as‐deposited coatings, additional diffraction peaks corresponding to zinc hydroxide (ICDD 48‐1066) were detected after immersion, indicating the formation of corrosion products on the sample surface. The appearance of Zn(OH)_2_ confirms the interaction of the zinc substrate with the electrolyte despite the presence of the hydroxyapatite‐based coating.

The diffraction patterns of Zn‐HA and Zn‐HA‐Ag samples exhibited similar phase compositions after immersion. In both cases, the dominant phases remained Zn together with corrosion products identified as Zn(OH)_2_. Only minor changes in the diffraction patterns were observed with increasing immersion time from 2 to 8 h, suggesting relatively stable corrosion product formation during the investigated exposure period.

For the Zn‐HA‐Ag samples, reflections assigned to AgZn_3_ and AgZn intermetallic phases remained detectable after immersion. No additional crystalline silver‐containing corrosion products were identified by XRD. The bare Zn sample analysed after 8 h of immersion exhibited diffraction peaks corresponding predominantly to Zn and ZnO. In contrast, Zn(OH)_2_ was detected only on the coated samples under the investigated conditions.

### 3.5. Platelet Adhesion Behaviour

**FIGURE 14:**
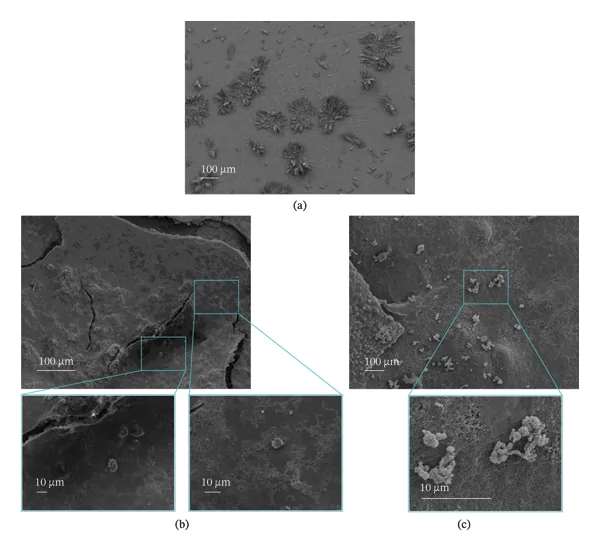
SEM images of adhered platelets on the surface of (a) Zn substrate and (b), (c) Zn‐HA‐Ag sample.

After exposure to platelet‐rich plasma, SEM analysis revealed no visible adherent platelets on the surface of bare zinc. The surface was predominantly covered with crystalline deposits originating from chemical species present in PBS and plasma. In contrast, the Zn surface coated with HA‐Ag exhibited increased platelet adhesion (Figure 14). Adherent platelets were observed either as isolated spherical cells or in small clusters. Most of the observed platelets retained a rounded morphology without pronounced pseudopodia formation, which may suggest limited platelet activation under the tested conditions.

### 3.6. Inhibition Effects Observed in the Disc Diffusion Assay

**FIGURE 15:**
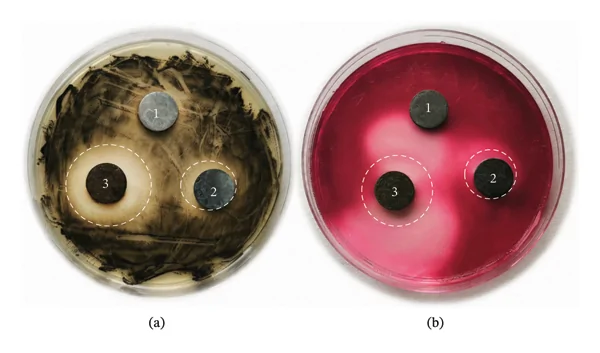
Disc diffusion test results for Zn‐based samples: (1) bare Zn, (2) Zn–HA and (3) Zn‐HA‐Ag, tested against (a) *Staphylococcus aureus* CCM 4223 and (b) *Escherichia coli* CCM 3954.

The disc diffusion assay revealed clear differences in the inhibition zone formation of the tested samples against *Escherichia coli* CCM 3954 and *Staphylococcus aureus* CCM 4223 (Table 5, Figure 15). The disc diffusion test was performed on Zn, Zn‐HA and Zn‐HA‐Ag samples. Bare Zn discs did not produce any visible inhibition zones, indicating the absence of detectable diffusion‐mediated antibacterial effects under the tested conditions. In contrast, the Zn‐HA samples produced moderate inhibition zones against both bacterial strains. For *E. coli*, the inhibition zone diameter reached 15.26 ± 0.64 mm, while a larger zone diameter of 17.57 ± 0.53 mm was observed for *S. aureus*. The relatively small inhibition widths measured from the disc edge suggest limited diffusion of the active species into the agar medium.

**TABLE 5:** Inhibition zone measurements of Zn disc samples from the disc diffusion method.

	Zn	Zn‐HA	Zn‐HA‐Ag
*E. coli CCM 3954*			
Inhibition zone diameter (mm)	0	15.26 ± 0.64	22.25 ± 1.28
Inhibition zone width from disc edge (mm)	0	1.13 ± 0.32	4.25 ± 1.04

*S. aureus CCM 4223*			
Inhibition zone diameter (mm)	0	17.57 ± 0.53	26.27 ± 1.19
Inhibition zone width from disc edge (mm)	0	1.75 ± 0.29	6.00 ± 0.89

Among all tested materials, the Zn‐HA‐Ag samples exhibited the most pronounced inhibition effect in the disc diffusion assay. For *E. coli*, the inhibition zone diameter was 22.25 ± 1.28 mm, with a corresponding inhibition width of 4.25 ± 1.04 mm. An even higher sensitivity was observed for *S. aureus*, where the inhibition zone diameter reached 26.27 ± 1.19 mm and the inhibition width from the disc edge was 6.00 ± 0.89 mm. These findings demonstrate that the Zn–HA–Ag samples produced larger diffusion‐mediated inhibition zones under the disc diffusion assay conditions. Overall, a stronger inhibitory effect was consistently observed against *S. aureus* compared to *E. coli*. This difference may be related to variations in bacterial cell wall structure, as the outer membrane present in Gram‐negative *E. coli* may act as an additional barrier limiting the penetration of antibacterial agents.

Besides that, the irregular, noncircular shape of the inhibition zones observed for the *E. coli* strain may be associated with the nonuniform diffusion of released antibacterial species, most likely Ag^+^ ions, into the agar medium. Local variations in released species concentration, agar hydration, surface contact and bacterial growth dynamics can lead to asymmetric inhibition patterns, as reported in the literature [53].

## 4. Discussion

Building upon our previous work [45], which focused on optimising the electrochemical deposition of hydroxyapatite on zinc substrates, the present study addressed the insufficient understanding of how silver addition affects Ag‐containing hydroxyapatite coatings when applied to highly reactive biodegradable zinc, where the substrate itself may influence the resulting properties of the coating system. To address this question, silver was introduced into a previously optimised hydroxyapatite deposition system while maintaining identical deposition conditions, enabling a systematic evaluation of its influence on the resulting coating–substrate system. The obtained results demonstrate that silver addition modified the surface chemistry, morphology, corrosion behaviour, tribological response and inhibition effects observed in the disc diffusion assay.

The optical microscopy analysis of the zinc substrate prepared by cold‐pressing and subsequent sintering showed the presence of visible striping on the surface, characterised by alternating lighter and darker bands. This striping occurred during the cold‐pressing process, likely due to the uneven distribution of Zn powder grains, which resulted in regions with varying density and structure. The uneven density and the presence of the micropores between Zn grains can increase the effective surface area of the sample and provide nucleation and anchoring sites that promote mechanical interlocking of the coating. Such micro‐roughness can reduce the risk of mechanical peeling and enhances the adhesion strength of the HA coating [54]. As evidenced by AFM results, the surface roughness and morphology were changed after the deposition of HA‐Ag, confirming the successful formation of the coating. An increase in the roughness parameter is related to the growth pattern of HA‐Ag during electrochemical deposition. SEM showed that this process leads to branch‐like structures composed of agglomerated particles forming globular clusters that result in a porous dendritic morphology. The higher roughness of the Zn‐HA‐Ag surface can enhance protein and cell bonding on the implant. The adhered cells allow the creation of a direct interfacial bond between bone and implant, which improves its stability compared to smooth implants [55].

The chemical composition of the sample surfaces was investigated using EDX, FTIR, XRD and XPS, providing complementary information on the elemental, molecular and crystalline characteristics of the zinc substrate and deposited coating. The EDX analysis of the bare zinc revealed the presence of oxygen, originating from ZnO formed due to zinc oxidation upon exposure to air. According to the observations of Ananth et al. [56], the presence of ZnO on the surface of the zinc substrate can lead to increased adhesion strength of the hydroxyapatite coating. Hydroxyl (OH^−^) groups on the oxidised Zn surface can act as nucleation sites, promoting the binding of PO_4_^3−^ and Ca^2+^ ions from the electrolyte during electrochemical deposition.

In the case of the zinc sample coated with silver‐containing hydroxyapatite, the detected presence of Ca, P and O is characteristic of calcium phosphate ceramics. In addition, Zn and Ag were consistently detected in both area and point EDX analyses performed on the dendritic coating structures, confirming the presence of Zn‐ and Ag‐containing species within the coating. The point analysis showed the presence of Zn, Ag and O, which could be associated with the formation of metal oxides on the sample surface. The detected carbon on the surface of the coated sample originated from atmospheric CO_2_ [57], which dissolved into the electrolyte solution during the electrochemical deposition of the coating and the formed CO_3_^2−^ ions subsequently substituted PO_4_^3−^ ions within the hydroxyapatite structure. The presence of carbonate groups was further confirmed by additional spectroscopic analyses. The formation of CO_3_^2−^ groups occurs via the following reactions [58]:

(2) CO2+H2O⟶H2CO3,

(3) H2CO3+OH−⟶ HCO3−+H2O,

(4) HCO3−+OH−⟶ CO32−+H2O.

FTIR analysis of the coated sample revealed the characteristic functional groups of the hydroxyapatite. The band at 1440 cm^−1^ corresponds to the asymmetric stretching vibrations (*ν*_3_) confirming the presence of CO_3_^2−^ ions. The position of this band within the wavenumber range of 1545–1450 cm^−1^ is associated with the B‐type substitution, in which CO_3_^2−^ ions replace PO_4_^3−^ ions [42]. A prominent sharp band at 1025 cm^−1^ and an additional band at 565 cm^−1^ correspond to the stretching vibrations of PO_4_^3−^, which align with previously reported literature data [59].

The literature on undoped hydroxyapatite indicates the presence of a broad band in the region of 3458 cm^−1^, attributed to the stretching vibrations of OH^−^ groups [60]. However, this band is absent in the FTIR spectrum of Zn‐HA‐Ag. A similar disappearance of the OH^−^ band in silver‐doped hydroxyapatite was observed by Ruíz‐Baltazar et al. [61]. This absence may be attributed to structural modifications induced by silver incorporation, suggesting that Ag^+^ ions might be integrated into the hydroxyapatite lattice. According to previous studies [38], these structural changes may be attributed to the partial substitution of Ca^2+^ ions by Ag^+^ ions.

The bands observed at 2919 and 2850 cm^−1^, forming a doublet, correspond to the stretching vibrations of C‐H bonds. These bands likely originate from ethanol or acetone residues, which were used for sample cleaning before the deposition process [62]. The absorption band at 539 cm^−1^ corresponds to ZnO, as surface oxidation of Zn readily occurs during processing and exposure to the atmosphere [63].

The crystallographic structure and phase composition of the prepared coating were analysed by X‐ray diffraction. XRD analysis of the Zn substrate confirmed the characteristic reflections of metallic zinc and ZnO. Analysis of coated samples revealed the characteristic diffraction patterns for HA corresponding to the ICDD standard, which indicates that this is the predominant phase in the ceramic coating. Additionally, in the Zn samples coated with HA and HA‐Ag, reflections corresponding to hydroxyapatite were of relatively low intensity, indicating the formation of coatings with low crystallinity and low thickness. The presence of characteristic HA reflections in both cases, Zn coated with HA–Ag after thermal alkali treatment and the untreated HA and HA–Ag powders obtained from the electrolyte after electrochemical deposition, demonstrates that the conditions used in this study promoted the direct formation of hydroxyapatite without the need for post‐treatment. Based on the literature, the reduction of HA peak intensity in HA‐Ag can be attributed to the Ag incorporation to the HA structure, which caused alterations in HA crystal structure leading to the lower crystallinity. The incorporation of Ag might affect the nucleation and growth of crystalline HA. Similar observations have been reported in previous study, where Ag incorporation resulted in lattice distortions [64]. Lower crystallinity can be advantageous for orthopaedic applications, as it typically increases the dissolution rate of HA and so supports bioresorption and stimulates osteointegration. Considering that analysis in the HA‐Ag coating revealed reflections characteristic for silver in the hexagonal face‐centred cubic (FCC) structure, we can assume that silver is in the coating present also in the form of particles.

Reflections of intermetallic phases AgZn and AgZn_3_ determined in the Zn‐HA‐Ag samples indicate the incorporation of Ag to the zinc matrix during the electrochemical deposition of the coating. This phenomenon can be explained by the reaction between the Zn and Ag^+^ ions by rinsing of the Zn sample to the electrolyte solution with AgNO_3_ during electrochemical deposition. Considering the noble character of silver with more negative *E* ^0^, the substitution reaction occurred

(5) Zns+22Agaq+⟶ Znaq2++Ags.

During this reaction, the zinc is oxidised and dissolved to the solution and silver is reduced. As silver has similar atomic radii as zinc (0.144 and 0.138 nm, respectively), silver easily filled empty places in the metallic lattice of the zinc.

This observation is in agreement with the EDX results, which revealed a relatively high Zn content in the HA‐Ag coating. The presence of Zn within the coating system may be associated with the oxidation of the zinc substrate during electrodeposition and the subsequent incorporation of Zn‐containing species into the deposited layer. The Zn‐doped HA can improve antibacterial properties [65] of the material and enhance osteogenesis by supporting osteogenic proliferation [66].

The XPS survey spectra confirmed the presence of the same elements on the sample surfaces as detected by EDX analysis. This observation indicates partial oxidation of the zinc surface due to exposure to ambient air humidity. Figure 8a presents Zn 2p spectra, where all samples exhibit two characteristic spin–orbit components corresponding to Zn 2p^3/2^ at 1022 eV and Zn 2p^1/2^ at 1045 eV. As the Zn 2p region does not allow a reliable distinction between metallic Zn (Zn^0^) and oxidised Zn (Zn^2+^), Zn LMM Auger spectra were obtained (Figure 8b). The comparison of the Zn LMM spectra for coated and uncoated samples revealed that the spectrum of uncoated Zn contains, in addition to the main kinetic energy peak at 988 eV, an additional contribution at 993 eV corresponding to Zn^0^ [67, 68]. The Auger signal of Zn LMM also overlaps with the Na KL1 signal at approximately 989 eV, as shown in Figure 8b, which makes more precise quantification of Zn signal very difficult. The decrease of Zn content in Zn‐HA and Zn‐HA‐Ag confirms the successful coating (Table 1). The bare Zn sample exhibited a Zn 2p atomic concentration of 14.9 at. %, which decreased markedly to 1.6 at. % for the Zn‐HA sample. In the case of the Zn‐HA‐Ag sample, the Zn content is slightly higher (5.8 at. %, Table 2).

The higher Zn^2+^ signal intensity observed for Zn‐HA‐Ag compared to Zn‐HA indicates a greater contribution of Zn‐containing species within the analysed surface region. This observation is consistent with the EDX results, which also revealed a higher Zn content for the Zn‐HA‐Ag coating compared to Zn‐HA. Together, these findings suggest an increased presence of Zn‐containing species in the Zn‐HA‐Ag coating system. However, the employed analytical techniques do not provide sufficient information to determine their precise chemical or structural form.

Figure 8c shows Ca 2p spectra of Zn‐HA and Zn‐HA‐Ag samples. In both cases, two peaks were observed at binding energies of approximately 347.1 and 350.6 eV, corresponding to the Ca 2p^3/2^ and Ca 2p^1/2^ components, respectively. P2p signal at ca 133 eV for both coated samples confirms the presence of PO_4_^3-^.

According to Abdullin et al. [69], the O 1s peak at ca 530 eV in Zn corresponds to O^2-^ of ZnO, while the peak at 531.8 eV can be associated with OH^−^ groups of hydroxylated ZnO present on the surface. Zn sample also exhibits high content of adventitious carbon on the surface, which is also reflected in the O1s at ca 531.8 eV corresponding to organic oxygen (Figure 9a). In the case of coated samples, O1s shows strong peak at ca 531.4 eV. The dominant signal at 531.4 eV can be assigned to overlapping contributions from OH^−^, PO_4_^3−^ and CO_3_^2−^ groups in hydroxyapatite, which is in good agreement with previously published data [70]. Negrila et al. [71] reported that O 1s binding energies in the range of 531–531.5 eV can be attributed to phosphates. The binding energy of hydroxyl ions has been reported at 531.8 eV by Gagiotti et al. [72] and at 531.2 eV by Kawabe et al. [73], both of which are close to the values obtained in this study. Kačiulis et al. [74] confirmed that in hydroxyapatite, the main O 1s component at ∼531.6 eV arises from OH^−^, PO_4_^3−^ and CO_3_^2−^ groups, which cannot be reliably separated by peak fitting. O1s at ca 530.6 eV can be attributed to some carbonyls on the surface and O1s at ca 532.8 eV to C‐O groups [75]. These findings are consistent with the results obtained from EDX, FTIR and XRD analyses.

In the C 1s HR spectra of both samples, the main peak at 284.8 eV is attributed to C‐C and C‐H bonds [76]. C1s at ca 288.9 eV showed small contribution from carboxylic groups (O‐C=O or COOH) for Zn sample (3.7 at. %), while after coating, we detected signal at ca 289.6 eV corresponding to CO_3_^2−^ [67, 68]. This carbonate is most probably related to the CaCO_3_. Ca/P ratio in HA is roughly 1.67, so in both cases (Zn‐HA and Zn‐HA‐Ag), we have excess of Ca of about 5–6 at. % (calculated from Table 1), which nicely correlate with the number of carbonates (ca 7 at. %, Table 2). The presence of the carbonate group was also confirmed by FTIR analysis.

**FIGURE 16:**
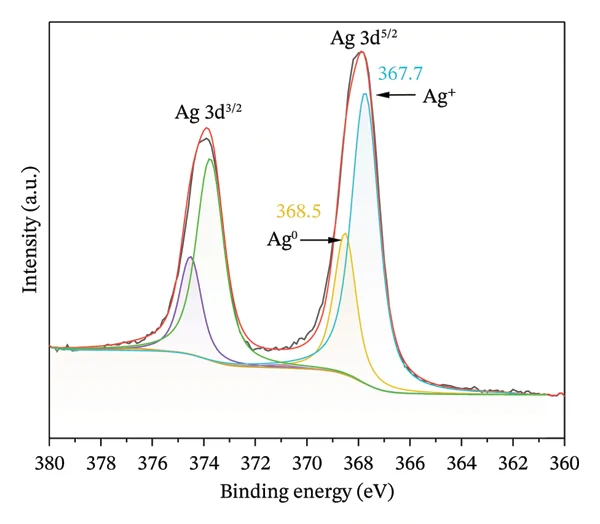
High‐resolution XPS spectra of Ag 3d region.

In Ag 3d, we identified two contributions (Figure 16). The Ag 3d^5/2^ component centred at 367.7 eV was attributed to oxidised Ag^+^ species, whereas the component centred at 368.5 eV was assigned to metallic silver [67, 68]. These results confirm the presence of Ag‐containing species in different oxidation states in the Zn‐HA‐Ag sample. However, XPS does not provide direct information about their crystallographic location; therefore, the substitution of Ca^2+^ by Ag^+^ within the HA crystal lattice cannot be confirmed based on the present data. In addition to Ag^+^, a small contribution from metallic silver was detected, which is consistent with XRD results and indicates the presence of silver also in the form of metallic particles. The low signal of metallic silver can also be caused by the partial surface oxidation of these particles.

Overall, the XPS analysis demonstrates that electrochemical deposition led to the formation of a Ca‐P‐based layer on the Zn substrate. The combined XPS, EDX and XRD results further confirm the presence of silver‐ and zinc‐containing species within the coating system. XPS analysis revealed the coexistence of metallic and oxidised silver species on the coating surface, which, together with the XRD results, suggests that silver may be present within the coating, at least partially, in metallic form.

The performance of biodegradable orthopaedic implants depends not only on their biological compatibility but also on how their surface properties influence degradation and mechanical stability. Coatings, such as hydroxyapatite on zinc, can modify the initial interactions with the surrounding environment and affect wear behaviour. During the initial running‐in phase, microstructural adjustments, surface smoothing and the formation or removal of tribofilms occur, which collectively influence the material pair’s long‐term wear resistance. Understanding these early interactions is essential, as they provide insights into how surface coatings, such as hydroxyapatite on zinc, can enhance or limit durability under physiological conditions.

The ‘negative wear’ observed for the Zn substrate during the initial friction cycles may be associated with temporary material accumulation or transfer within the contact region rather than actual negative material loss. Given the hardness mismatch between zinc (0.3–0.4 GPa [77]) and the stainless steel counterbody (2.26 GPa [78]), transfer or redistribution of material from the softer Zn surface provides a physically plausible explanation for this response. However, the possible contribution of local surface deformation to the recorded response should also be considered.

Although coated Zn samples exhibit higher initial penetration depth than the uncoated substrate, this behaviour may indicate the sacrificial function of the coating. During sliding, the fragmentation and redistribution of the ceramic layer may occur, potentially forming a protective debris shield that could limit the deformation and suppress the release of metallic artefacts, which is of particular concern in orthopaedic applications [79, 80]. However, the occurrence and role of these processes in the present system cannot be determined from the penetration depth profile alone.

The penetration depth profile of Zn‐HA‐Ag reveals the onset of quasi‐negative wear after approximately 100 sliding cycles, suggesting a temporary change in the contact response of the coated surface. This delayed transition is consistent with previous findings, where HA coatings postponed substrate participation until roughly 50 cycles [45]. Post‐test wear‐track characterisation and wear volume quantification were not experimentally performed in this study. However, based on the literature, such behaviour may underscore the early‐stage shielding capacity of HA‐based coatings and highlights the importance of coating integrity during the running‐in phase.

The observed tribological performance of the Zn‐HA‐Ag system cannot be attributed solely to coating thickness applied in this study. Previous studies have reported that Ag addition may alter the microstructure and mechanical properties of HA‐based materials [81]. Such changes may contribute to differences in the response of Ag‐containing HA materials during sliding.

The obtained coefficients of friction, which express the ratio of the frictional force resisting the motion of two surfaces in contact to the normal force pressing them together, further demonstrate different responses of the coated and uncoated surfaces. After approximately 20 cycles, the Zn‐HA‐Ag sample exhibited lower CoF values than the Zn substrate for the remainder of the test. The gradual increase in the CoF of the Zn substrate may be associated with progressive changes in the surface and contact conditions during sliding. As the initial running‐in response progresses, these changes can result in increased friction.

Taken together, the Zn‐HA‐Ag sample exhibited a different tribological response from uncoated Zn under the applied reciprocating sliding conditions. After approximately 20 cycles, the coated sample maintained lower CoF values, and after approximately 110–120 cycles, its penetration depth remained lower than that of uncoated Zn. At the end of the test, Zn‐HA‐Ag exhibited both a lower CoF and a lower penetration depth, indicating a more favourable friction and penetration depth response of the coated system under the specific test conditions.

The degradation behaviour of the prepared samples was evaluated electrochemically using OCP measurements followed by potentiodynamic polarisation and EIS in Hank’s solution simulating body fluids. During OCP stabilisation, the Zn‐HA‐Ag sample exhibited less stable potential evolution compared to bare Zn, which may be associated with the more complex electrochemical behaviour of the coated surface. While bare Zn showed gradual stabilisation that may be related to surface passivation, the behaviour of the coated sample could additionally be influenced by coating heterogeneity, local surface defects and possible interfacial electrochemical interactions.

The polarisation measurements showed that the Zn‐HA‐Ag‐coated samples exhibited lower calculated corrosion current density and corrosion rate values together with increased polarisation resistance compared to bare Zn. However, the simultaneous shift of OCP and corrosion potential towards more negative values suggests that the corrosion behaviour of the coated system cannot be interpreted solely as a simple passive barrier effect. Possible local galvanic interactions between Zn and silver‐containing species present within the coating, particularly at pores or coating defects observed by SEM, may also contribute to the overall electrochemical response of the system. Similar behaviour has been discussed in previous studies dealing with multifunctional coatings on biodegradable metallic substrates [51].

To obtain further insight into the coating behaviour of the studied samples and to verify the trends observed from PDP measurements, EIS was performed. The EIS results were generally consistent with the polarisation measurements. The Nyquist plots revealed a shift of the impedance response towards higher *Z* ^′^ values after silver incorporation into the hydroxyapatite coating, indicating a modification of the electrochemical behaviour of the coated surface. Equivalent circuit fitting of the coated samples further showed an increase in the resistive contribution associated with the coating layer (*R*_*f*_) from 29.92 ± 0.74 Ω for Zn‐HA to 58.48 ± 0.70 Ω for Zn‐HA‐Ag. Similar increases in coating resistance have previously been associated with reduced electrolyte penetration and improved barrier properties of hydroxyapatite‐based coatings [82]. This observation suggests that the overall impedance response was predominantly influenced by changes in coating structure and electrolyte accessibility rather than by the presence of silver itself.

In addition, the exponent of the coating‐related constant phase element (*n*_*f*_) increased from 0.88 ± 0.01 to 0.93 ± 0.01 after Ag incorporation. Values of *n* approaching unity indicate a response closer to that of an ideal capacitor and are commonly associated with a more uniform electrochemical behaviour of the surface layer. The lower *Q*_*f*_ value observed for Zn‐HA‐Ag may further indicate a reduced effective electrochemically active area exposed to the electrolyte. These observations are in agreement with the lower corrosion current density and corrosion rate obtained from polarisation measurements.

At the same time, the EIS results also suggest that the electrochemical behaviour of the coated system cannot be explained solely by the formation of a simple protective barrier. The decrease of *n*_int_ from 0.82 ± 0.01 for Zn‐HA to 0.68 ± 0.02 for Zn‐HA‐Ag indicates a more heterogeneous interfacial response after silver incorporation. This behaviour may be associated with the heterogeneous distribution of silver‐containing phases, coating defects and pores observed by SEM, as well as local electrochemical interactions occurring at the coating/substrate interface. At sites where the electrolyte reaches the underlying substrate through pores or coating defects, the presence of silver‐containing species may lead to locally different electrochemical behaviour. This may contribute to the increased heterogeneity of the interfacial response reflected by the lower *n*_int_ value.

The postcorrosion XRD analysis revealed the formation of similar crystalline corrosion products on both coated systems. Zn(OH)_2_ was detected after immersion in Hank’s solution for both Zn‐HA and Zn‐HA‐Ag samples. The similarity of the corrosion products formed on Zn‐HA and Zn‐HA‐Ag indicates that the differences observed in the electrochemical measurements cannot be directly attributed to the formation of distinct crystalline corrosion phases. Instead, the EIS results suggest that silver incorporation primarily influences the electrochemical response of the coating/electrolyte system, as reflected by the increased coating resistance and changes in the parameters of the coating‐related constant phase element. Together with the polarisation results, these findings indicate that the effect of silver is associated with modifications of the electrochemical behaviour of the coated surface rather than with the formation of different crystalline corrosion products.

As platelet adhesion represents an important aspect of blood–material interactions, platelet adhesion behaviour on the prepared samples was evaluated using a platelet adhesion assay. Compared to bare Zn, increased platelet adhesion was observed on the HA‐Ag‐coated surface, which may be related to changes in surface morphology and roughness after coating deposition. The adherent platelets were predominantly present as spherical cells or small clusters without pronounced pseudopodia formation, which may suggest limited platelet activation under the tested conditions. However, it should be noted that the present evaluation was limited to qualitative SEM observations and does not represent a comprehensive assessment of thrombogenicity or hemocompatibility. Moreover, this assay does not provide information on the cytocompatibility of the coating or on the biological effects of Ag‐ and Zn‐containing species potentially released during degradation. Quantitative ion release measurements and cytocompatibility testing are therefore required before the biological safety of the coating can be assessed.

The diffusion‐mediated inhibition effects of the prepared samples were tested by the disc diffusion test against *S. aureus* and *E. coli*. Not surprisingly, the inhibition zone formation of the bare Zn substrate was negligible under the tested condition**s.** Similar observations have been reported in previous studies, where pure metallic zinc exhibited limited or no inhibition zone formation in diffusion‐based tests, primarily due to insufficient release of Zn^2+^ ions into the surrounding medium [53, 83]. Upon exposure to aqueous environments, metallic Zn is prone to rapid surface passivation, resulting in the formation of a native layer composed of zinc oxide and zinc hydroxide (ZnO/Zn(OH)_2_) [83]. This passive layer effectively suppresses further corrosion and significantly hinders the release of biologically active Zn^2+^ species from the surface. Consequently, the diffusion of antibacterial zinc ions into the agar medium is strongly limited, leading to negligible inhibition effects in disc diffusion assays despite the intrinsic antimicrobial potential of zinc ions.

In contrast, the Zn sample coated with a hydroxyapatite (HA) layer exhibited measurable inhibition zones against both *E. coli* and *S. aureus* **.** Zn‐incorporated hydroxyapatite systems have been widely investigated due to their combined bioactivity and antibacterial potential [84–86]. In the present system, this behaviour may be associated with the presence of Zn‐containing species detected by EDX and XPS analyses and their possible association with the HA coating layer. Compared to the bare Zn substrate, the HA‐coated samples exhibited larger inhibition zones in the disc diffusion assay, which may suggest altered diffusion behaviour of released Zn‐containing species into the surrounding agar medium.

Finally, the Zn‐HA‐Ag system exhibited the largest inhibition zones among the tested samples, with values significantly higher than those observed for Zn‐HA alone. Silver‐containing hydroxyapatite coatings are widely discussed in the literature due to their potential antibacterial effects against both Gram‐positive and Gram‐negative bacteria [38, 87]. Previous studies have also suggested that the simultaneous presence of Ag‐ and Zn‐containing species in HA coatings may contribute to increased inhibition effects compared to single‐ion systems [88]. More recently, hierarchical silver‐containing hydroxyapatite coatings deposited on TiO_2_ nanotubes were shown to produce inhibition zones against *E. coli*, illustrating that coating architecture and layer configuration may also influence the observed antibacterial response [89].

In the present Zn‐HA‐Ag samples, the larger inhibition zones observed in the disc diffusion assay may be associated with the presence of released Ag‐ and Zn‐containing species. According to the literature, released Zn‐containing species may contribute to membrane destabilisation and metabolic disruption [85], while Ag^+^ ions may interact with thiol‐containing enzymes and bacterial DNA [90, 91]. However, it should be noted that the present study did not include direct mechanistic antibacterial analyses or ion release measurements capable of conclusively confirming these mechanisms in the investigated system.

Based on the obtained results and comparison with the previously reported Zn‐HA coating [45], silver was introduced into an already optimised electrochemical deposition system while keeping the deposition parameters unchanged. This approach enabled a direct comparison between both coatings and allowed the influence of silver‐containing species on the resulting surface characteristics and functional properties to be evaluated. For clarity, Table 6 summarises the principal physicochemical, electrochemical, tribological and biological characteristics of both systems and highlights the overall effect of silver addition.

**TABLE 6:** Comparative summary of the effect of silver addition on the physicochemical, corrosion, tribological and biological properties of electrochemically deposited hydroxyapatite coatings on zinc substrates.

Property	Zn‐HA	Zn‐HA‐Ag	Effect of Ag addition
*Surface and structural properties*			
Surface morphology	Globular CaP aggregates [45]	Porous dendritic‐globular structure	↑ Structural complexity and porosity
Surface roughness	Lower (*R*_*a*_ = 47.3 nm) [45]	Higher (*R*_*a*_ = 65.1 nm)	↑ Surface roughness
Chemical composition	CaP coating containing Zn species [45]	CaP coating containing Zn and Ag species	Introduction of Ag‐containing species
Phase composition	HA dominant phase	HA + Ag + AgZn/AgZn_3_ phases	Formation of Ag‐containing phases

*Corrosion behaviour*			
Corrosion rate	0.48 mmpy [45]	0.42 mmpy	No significant change
Polarisation resistance	252.03 Ω [45]	401.31 Ω	↑ Polarisation resistance
Coating resistance (*R*_*f*_)	29.92 ± 0.74 Ω	58.48 ± 0.70 Ω	↑ Coating resistance
Dominant corrosion product after immersion	Zn(OH)_2_	Zn(OH)_2_	No significant change

*Tribological properties*			
Running‐in behaviour	Transition to quasi‐negative wear after ∼50 cycles [45]	Transition to quasi‐negative wear after ∼100 cycles	Shift of the observed transition in the penetration depth response from approximately 50–100 cycles

*Biological properties*			
Platelet adhesion and morphology	Solitary adhered platelets with limited activation [45]	Solitary spots and small groups of adhered platelets with limited activation	Comparable platelet morphology with altered distribution
Bacterial inhibition against *E. coli*	Inhibition zone diameter 15.26 ± 0.64 mm	Inhibition zone diameter 22.25 ± 1.28 mm	↑↑ Inhibition zone diameter
Bacterial inhibition against *S. aureus*	Inhibition zone diameter 17.57 ± 0.53 mm	Inhibition zone diameter 26.27 ± 1.19 mm	↑↑ Inhibition zone diameter

*Note:* The Zn‐HA and Zn‐HA‐Ag samples were tested within the same experimental series, while the Zn‐HA polarisation, tribological and platelet adhesion data were previously reported in Ref. [45]. Ra‐average surface roughness. Rf‐coating resistance. Jcorr‐corrosion current density. Ecorr‐corrosion potential.

Taken together, the results demonstrate that silver addition modifies the properties of electrochemically deposited hydroxyapatite coatings at several levels, from surface characteristics and corrosion behaviour to tribological and biological responses.

## 5. Conclusions

The present study demonstrated the successful electrochemical deposition of an Ag‐containing calcium phosphate coating directly on a zinc substrate using a previously optimised deposition system. Combined XRD, FTIR, EDX and XPS analyses confirmed the formation of a calcium phosphate–based coating containing silver and zinc species. Electrochemical measurements indicated a reduction in corrosion current density and corrosion rate together with an increase in polarisation resistance compared to bare zinc. The coating also improved the tribological response of the zinc substrate under the applied testing conditions. Under the disc diffusion assay conditions, the Ag‐containing coating produced larger inhibition zones against both *E. coli* and *S. aureus* than bare Zn and Zn‐HA samples. Platelet adhesion experiments showed predominantly spherical platelet morphology, suggesting limited platelet activation under the investigated conditions. Furthermore, the formation of AgZn and AgZn_3_ intermetallic phases indicates that silver addition affected not only the coating itself but also the overall coating–substrate system. Overall, the results provide insight into the relationship between coating composition, surface characteristics, corrosion behaviour, tribological response and preliminary biological performance of Ag‐containing calcium phosphate coatings on biodegradable zinc substrates. Future studies should focus on detailed biological evaluation including cytocompatibility testing, ion release behaviour and optimisation of coating microstructure and homogeneity to further improve the performance of such multifunctional biodegradable systems.

## Author Contributions

Ivana Mojžišová: conceptualisation, methodology, investigation, formal analysis, data curation, visualisation and writing–original draft; Radka Gorejová: conceptualisation, methodology, formal analysis, supervision, writing–review and editing and funding acquisition; Evghenii Harea, Tibor Sopčák and Matej Mičušík: investigation, formal analysis and writing–original draft; Pavlina Jevinová: investigation, data curation and formal analysis; Kadir Özaltin: investigation and formal analysis; and Renáta Oriňaková: supervision and writing–review and editing.

## Funding

This work was funded by the EU NextGenerationEU through the Recovery and Resilience Plan for Slovakia under project ZETA no. 09I03‐03‐V04‐00010.

## Conflicts of Interest

The authors declare no conflicts of interest.

## Data Availability

Data used to support the findings of this study are included in the article. If additional data or information is needed, these are available from the corresponding author upon request.
